# Mental health disabilities and human rights protections

**DOI:** 10.1017/gmh.2015.18

**Published:** 2015-10-01

**Authors:** G. Szmukler, M. Bach

**Affiliations:** 1 Institute of Psychiatry, King's College London, UK; 2 IRIS – Institute for Research and Development on Inclusion and Society, Kinsmen Building, York University, North York, Ontario, Canada

**Keywords:** Human rights, mental disability, mental disorder, mental health law, policy & systems

## Abstract

**Background.:**

Around the world, reports regularly expose persistent and systemic human rights violations of patients in mental health services and facilities, and of those who are unable to access needed supports. A number of factors contribute – political will; the range and quality of services available; public and professional attitudes to mental health; stigma; health professionals’ training and expertise; and available resources.

**Methods.:**

This paper examines one of the main determinants, the legal framework. This sets the parameters for mental health policies and services and for applicable human rights norms and standards that can be realized in practice.

**Results.:**

We provide an overview of international human rights instruments in relation to mental health disabilities, and of the major human rights violations in this area. Key implications for mental health law reform are drawn with a particular focus on discrimination and coercive interventions. The major challenges posed by the UN Convention on the Rights of Persons with Disabilities (2006) are examined. Current mental health laws, to greater or lesser degrees, fail to meet the newly required standards. We discuss reforms based on ‘generic law’ and ‘legal capacity’ principles that seek to meet those standards.

**Conclusions.:**

We outline some emergent and promising examples of reform. The role of civil society and the importance of the standing of those with mental health disabilities in this process is noted.

## Introduction

Around the world human rights monitoring and media reports regularly expose persistent and systemic human rights violations of patients in mental health services and facilities, and of those who are unable to access needed supports. A complex of factors contribute – political will; the range and quality of services available; public attitudes; health profession ethics, training and expertise; and available resources. This paper focuses on one of the main determinants – the legal framework. This is an important element since it sets the parameters for mental health policies and services and for applicable human rights norms and standards to be realized in practice. It also establishes published standards to which states can be held to account. Finally, the legal framework affects the standing in society of people with mental health disabilities. The legal framework does not, of course, ensure that the practices it mandates will occur.

Legislative provisions are shaped by various international charters, conventions and covenants that give expression to fundamental values and principles. We examine these international commitments and how they might be drawn upon to reshape practice to address human rights violations experienced by many people with mental health disabilities.

The first part of the paper provides an overview of international human rights instruments and disability, and is followed by a brief discussion of the global context of human rights violations in the area of mental health. Key implications for mental health law reform are then described with a particular focus on discrimination and non-consensual or coercive interventions. The paper concludes with an outline of some emergent and promising examples of reform.

Our analysis is guided by the norms and provisions recognized in the UN Convention on the Rights of Persons with Disabilities (CRPD) (discussed in detail below) and by its approach to characterizing disability: ‘*Persons with disabilities include those who have long-term physical, mental, intellectual or sensory impairments which in interaction with various barriers may hinder their full and effective participation in society on an equal basis with others’.* ‘Include’ in the statement above allows for a non-exhaustive description of ‘disability’ that is not settled. We take the term to embrace those with a mental disorder or impairment that has persistence over time and who may therefore have a range of encounters with the mental health system, experience the effects of social stigma or remain without access to needed health supports.

## International human rights law and mental disability

What do we mean by ‘human rights’? At a philosophical level the concept of ‘human rights’, rights that are applicable by virtue of being human, and their foundational basis are controversial – that is, are they founded in ‘natural law’, in what are recognized as legally valid claims motivated by ethical demands (Sen, 2004), or in social practices that constitute particular understandings of freedom and dignity that are given authoritative legal recognition? See, for example, Martin (2013). Our approach starts with international rights instruments which, whatever the ultimate source of the rights they recognize, are accepted as legitimate norms to which the vast majority of states in the world are signatories. These include both UN and regional human rights instruments. The former include the Universal Declaration of Human Rights (1948) which together with the International Covenant on Civil and Political Rights (1966) and the International Covenant on Economic, Social and Cultural Rights (1966), form what is known as the ‘International Bill of Rights’. Regional instruments include the European Convention on Human Rights (1950), the African Charter on Human and Peoples’ Rights (1981), the European Convention for the Prevention of Torture and Inhuman or Degrading Treatment or Punishment (1987), and the American Convention on Human Rights (1978) (see WHO, 2010*a* for details). While mental illness or disabilities are rarely specifically addressed, the rights embodied in these instruments are taken to apply to all persons.

Recently, the human rights position of persons with ‘disabilities’ has been clearly specified through the CRPD adopted by the United Nations in 2006 (United Nations General Assembly, 2007). By the end of 2014 the CRPD had been signed by 159 states and ratified by 153. Noteworthy was the formal, active involvement of disabled people's organizations in the drafting and negotiations behind the CRPD. We shall take the rights in this Convention as our point of reference.

The overall purpose, stated in Article 1, is to ‘*promote, protect and ensure the full and equal enjoyment of all human rights and fundamental freedoms by all persons with disabilities, and to promote respect for their inherent dignity*’. The elimination of discrimination by ensuring that rights may be enjoyed ‘*on an equal basis with others*’ is a fundamental aim. The CRPD contains classic civil and political rights, such as the right to liberty (Article 14) to integrity of the person (Article 17), to freedom of expression (Article 21), to privacy (Article 22), to freedom from torture and inhuman treatment (Article 15), to equal recognition before the law (Article 12) and access to justice (Article 13). It also includes economic, social and cultural rights that have come to prominence more recently, including the right to home and family life (Article 23), to education (Article 24), to health (Article 25), and to habilitation and rehabilitation (Article 26). Some of these rights have been framed so as to have particular relevance to people with disabilities: rights to non-discrimination (Article 5), to independent living and community inclusion (Article 19), to work and employment (Article 27), to participation in cultural life (Article 30) and to be free from exploitation and abuse (Article 16).

Countries are placed under obligations to modify or abolish existing discriminatory laws, regulations and practices, as well as to provide programs to support CRPD rights (Article 4). These include, for example, a duty to provide training on disability issues to those involved in the administration of justice (Article 13), programs to recognize and combat exploitation (Article 16), to provide community support services (Article 19), to raise awareness of disability issues (Article 8) and to combat discrimination (Article 5).

The Convention establishes the UN Committee on the Rights of Persons with Disabilities, to which State Parties are to report periodically about their progress in its implementation. The Committee in turn publishes its observations about this progress. The CRPD requires governments to ensure that representatives of civil society, in particular persons with disabilities, are fully involved in this monitoring. State Parties signing the Optional Protocol, recognize the competence of the Committee to examine complaints from individuals; determinations are again public.

Depending on the jurisdiction, the CRPD may or may not be automatically domesticated into law upon ratification. In many common law countries (such as the UK) it is only incorporated into domestic law when directly legislated. Like any other international convention to which a state is party, it can, however, be referred to by courts and be used to interpret domestic law.

## The global context of mental disability and human rights violations

The WHO estimates that 450 million people worldwide experience mental, neurological or behavioural problems, but that the majority lack access to needed mental health care (World Health Organization, 2010*b*). Global mental health expenditures are not able to keep up with the need for support. A hugely disproportionate investment of resources is made in institutional and acute care compared to community-based services, resulting in the vast majority not accessing the supports they require. In developing and low-income countries prevalence trends are exacerbated due to socio-economic conditions while investments are minimal compared to responses to physical health needs [for example, the WHO (2010*b*) reports that in Uganda depression affects almost as many people as malaria, but draws only a fraction of the investment in response]. Moreover, in many lower-income countries the disproportionate investment in a few acute care facilities compared with community-based care is that much more extreme. These factors set a context for an increasing vulnerability of this group and systemic human rights violations.

We need not detail abuses here; they are hardly secret and copious documentation is available in reports from bodies such as the WHO, Mental Disability Advocacy Centre (http://www.mdac.info/en) and Human Rights Watch (http://www.hrw.org/). A useful summary by Drew *et al*. (2011) of human rights violations in 18 low- and middle-income countries reveals how pervasive they are. The most common are: exclusion and discrimination in the community; denial or restriction of employment; physical or sexual abuse; inability to access services; arbitrary detention; denial of opportunities for marriage and family life; lack of means to live independently; financial exploitation.

Entrenched stigma and discrimination underlie human rights abuses. Negative stereotypes are not only widespread among the general public; research also points to widespread stigmatizing beliefs and attitudes among mental health professionals, across regions, including Western industrialized countries (Thornicroft *et al*. 2009).

## Mental health law today: a global perspective

How do current mental health laws in countries around the world measure up as regimes, in principle at least, to protect against and address rights violations? The latest available data on such laws come from the WHO Mental Health Atlas 2011 (WHO, 2011). Legislation may cover a broad range of issues including access to mental health care and other services and their quality; admission to mental health facilities; consent to treatment; freedom from cruel, inhuman and degrading treatment; freedom from discrimination; the enjoyment of civil, cultural, economic, political and social rights; and, legal mechanisms to promote and protect human rights (e.g. review bodies to oversee admission to mental health facilities and treatment, monitoring bodies and complaints mechanisms).

In 2011 only 59% of the world's people lived in a country with *dedicated* mental health legislation. The numbers varied according to the country's level of income: 77% in high-income countries compared with only 39% in low-income countries. In 15% of countries with legislation, it was enacted before 1970; in 42% it was enacted or revised after 2005. Legal provisions relevant to mental health in *non-dedicated* legislation – covering, for example, welfare, disability, general health, discrimination – were present in 71% of countries. Six per cent of countries had no relevant legislation at all. These figures will have changed significantly following The People's Republic of China's adopting, for the first time, a specific mental health law in 2012. This adds another 20% of the world's population to those covered by specific legislation.

We suggest it is helpful to think about the legal protection of the human rights of persons with mental disabilities in terms of levels. We propose the following typology: *Level 0* is where there are no laws concerning those rights. *Level 1* is where there are relevant laws but where they are generally not observed in practice. (This may be due, for example, to lack of political will, lack of public interest, lack of resources – human and material – to enable their observance, because the laws are dated or otherwise unworkable, or the absence of a ‘voice’ of people with mental disabilities). *Level 2* is where there are relevant laws that are observed, certainly a major advance on Level 1, but where they themselves may fall short of the desired standard in protecting the rights of persons with mental health disabilities. Countries at *Level 3* have laws that are compliant with the UN CRPD and observed in policy and practice.

### Mental health law and discrimination

There are numerous examples of countries whose mental health regimes can be located to a greater or lesser degree at *Level 1*; for example, there may be unregulated use of involuntary treatment or the use of cruel, inhuman or degrading ‘treatments’ (see, for example, reports from the bodies mentioned above). This level of protection is clearly unacceptable and needs urgent remediation. Most of the remaining discussion will be concerned with *Levels 2* and *3*.

While not the findings of a systematic analysis of data across jurisdictions, given the evidence presented in the WHO atlas, we propose it is reasonable to assume that that existing dedicated mental health legislation in virtually all countries meets *Level 2* criteria, at best. The recent law in China is an example (Zhao & Dawson, 2014). Such legislation does not meet Level 3 standards because it is discriminatory. We discuss the reasons below.

## Towards a CRPD compliant approach to mental health law

What would a level 3 standard for mental health law look like, one that would be compliant with the CRPD? First, it would have to confront head on the matter of detention and involuntary treatment. Second, it would involve a complete reformulation of the capacity of persons with disabilities to make decisions and its legal recognition.

To take the first dimension, along with the general right to liberty, similar to that contained in other human rights instruments, the CRPD provides that ‘*the existence of a disability shall in no case justify a deprivation of liberty.*’ The Office of the UN High Commissioner for Human Rights has stated:

‘[48.] … Article 14, paragraph 1 (b) unambiguously states that ‘the existence of a disability shall in no case justify a deprivation of liberty’. …. As a result, unlawful detention encompasses situations where the deprivation of liberty is grounded in the combination between a mental or intellectual disability and other elements such as dangerousness, or care and treatment. Since such measures are partly justified by the person's disability, they are to be considered discriminatory and in violation of the prohibition of deprivation of liberty on the grounds of disability, and the right to liberty on an equal basis with others prescribed by article 14.†’ (UN General Assembly, 2009).

On this account, ‘mental disorder’ or ‘mental illness’, even if it comprises only one of a number of necessary criteria for involuntary detention, makes that set of criteria incompatible with Article 14.

With respect to the second dimension, the UN Committee on the Rights of Persons with Disabilities (2014), charged with issuing authoritative interpretations of key Articles in the CRPD, issued a General Comment on ‘Article 12: Equal recognition before the law’ which recognizes that all persons enjoy ‘*legal capacity*’ in all aspects of life on an ‘*equal basis with others*’. Article 12(3) also recognizes the obligation of States Parties to ensure access to the supports a person may require to exercise legal capacity. The General Comment defines the right to legal capacity as encompassing both the ability to ‘*hold rights and duties (legal standing) and to exercise those rights and duties (legal agency)*’, and makes clear that legal capacity and mental capacity are distinct concepts. However, unlike virtually all mental health and capacity law, the Committee finds that the CRPD requires that the existence of an impairment (including a physical, mental or sensory impairment), or a diagnosis, must never be grounds for denying legal capacity and imposing ‘*substitute decision making*’. The Committee states that supports in the exercise of legal capacity must be provided and that those involved ‘*must respect the rights, will and preferences of persons with disabilities and should never amount to substitute decision-making*’ (15).

In its ‘Concluding Observations’ on the reports of progress from 20 countries so far examined in implementing the CRPD, the Committee has concluded that states must ‘*take action to develop laws and policies to replace regimes of substitute decision-making by* ‘*supported decision-making,*’ *which respects the person's autonomy, will and preferences*’ (United Nations Committee on the Rights of Persons with Disabilities, 2015).

In light of the Committee's General Comment and Concluding Observations, we suggest that application of Articles 12 and 14 of the CRPD to mental health law results in two main standards to guide reform, and against which compliance can be measured:

(1) Is there a generic law for health care (and indeed social care) decision-making that applies equally to all treatment decisions – mental health or physical health?
(2) Do the legal provisions ensure individuals enjoy legal capacity without discrimination on the basis of disability – do they have access to the support they may require to exercise legal capacity in decision-making, in a manner that respects a person's will and preferences?

A brief overview of the implications for law reform of applying each of these standards follows. In short, it is our conclusion that if the two standards were applied, it would require a radical revision of current approaches to non-consensual interventions, in a manner we outline below.

### The generic law standard

The most obvious means of avoiding discrimination against persons with a mental health disability is by no longer differentiating between them and other persons. In other words, whatever the provisions that govern interventions that legitimately restrict or deprive a person of liberty, they should be generic and equally applicable to all persons, regardless of the type of diagnosis, mental or physical.

Conventional mental health law, virtually without exception, permits coercive interventions on the basis of two criteria: first, that the person has a ‘mental disorder’ (usually broadly defined), and second, that there is a substantial risk to the health or safety of the person, or to other persons. That is, a ‘status’ criterion and a ‘risk’ criterion. The law in China is a recent example.

In countries with well-developed health law people with ‘physical’ disorders – in contrast to the position of people with a ‘mental disorder’ – can make treatment decisions that may be seriously detrimental to their health or safety provided they have the relevant ‘decision-making capacity’. This is the case in most high-income countries and many others, including, for example, China under its Tort Liability Law 2009 (Zhao & Dawson, 2014). ‘Capacity’ is usually defined as the ability to understand, weigh and use information relevant to a decision and to communicate a decision. Thus the ‘autonomy’ or right to self-determination of persons with ‘mental disorder’ is not accorded the same respect as for all other persons receiving health care. This conventional approach to defining capacity is now in substantial question in light of the CRPD, as discussed below. Nonetheless, it is still the usual approach in health law.

People with mental health disabilities are furthermore subject to another form of discrimination – they are selectively singled out as liable to a form of preventive detention on the basis of putative risk alone. At any one time, the number of persons with a mental health disability who present a significant risk to others is a very small proportion of all persons who present such a risk. [As a guide, in England, 1.5% of serious violent offences are by patients with a ‘serious mental illness’ (Flynn *et al*. 2014).] However, only those with a ‘mental disorder’ are liable to be preventatively detained (usually, but not always, in a hospital) on the basis of perceived risk alone. For those posing an equal (or greater) risk to others but without a ‘mental disorder’, detention can only follow after the commission of an offence. This is clearly discriminatory. If preventive detention is to be allowed for those with a mental health disability solely on account of their risk to others, so should it be for everyone – or for no-one, including those with a mental health disability (Szmukler & Dawson, 2011).

A ‘status’ criterion of ‘mental disorder’ or something similar is clearly in direct contravention of Art 14 of the CRPD as we have noted earlier, making this kind of mental health law non-compliant.

What kind of legislative framework would thus be non-discriminatory? One of us has proposed what has been termed a ‘Fusion Law’ (Dawson & Szmukler, 2006). This is a single, generic statute, covering everyone, in any medical specialty, in any area of healthcare – and indeed social care – in any setting. That is, there would be no separate mental health or civil commitment law. In the initial formulation of the Fusion Law proposal, prior to adoption of the CRPD, an impairment in decision-making capacity would be required for non-consensual treatment in a similar manner to treatment decisions for those whose legal capacity is restricted under a ‘Mental Capacity’ Act, insofar as it is decision-specific, time-limited and reviewable. Further to an impairment of decision-making capacity the treatment would need to be in the person's ‘best interests’, and more clearly, according to the best interpretation of what the person would have chosen if they had retained capacity in the present circumstances (and, if appropriate, taking account of their present preferences).

A major advance of the proposed provisions is that the impaired capacity could be from any cause – a head injury, post-epileptic confusion, schizophrenia, confusion due to an adverse drug reaction or infection, Alzheimer's disease and so on. Involuntary treatment would cease when capacity has been re-established in a sufficiently stable manner. Furthermore, in this scheme, if these conditions were met and if treatment could be given effectively and safely, there would be no ethical objection to its being given in the community rather than in a hospital. Again, non-consensual treatment would end with the recovery of capacity.

Northern Ireland is preparing to adopt legislation along such principles, and would effectively become the first jurisdiction to meet this standard (Northern Ireland Executive, 2014). A modified version of the Fusion Proposal suitable for middle- and low-income countries has also been formulated (Szmukler *et al*. 2015).

### The non-discriminatory legal capacity standard

The CRPD establishes a new formula for the exercise of legal capacity in healthcare, property and personal care decisions. By de-linking mental capacity and legal agency, the Committee's interpretation makes clear that a person's cognitive and communicative abilities are no longer as singularly determinative in the constitution of decision-making capacity. A person's relevant abilities are part of the formula, but must be complemented by cognitive, communicative and interpretive assistance in the decision-making process as provided by designated supporters or third parties. This approach is referred to as ‘supported decision-making’ and legislative schemes to give it effect are being designed in a growing number of jurisdictions (Bach & Kerzner, 2010; Browning *et al*. 2015). Supported decision-making is an example of what is in CRPD terms a ‘reasonable accommodation’ – ‘a necessary and appropriate modification and adjustment not imposing a disproportionate or undue burden, where needed in a particular case, to ensure to persons with disabilities the enjoyment or exercise on an equal basis with others of all human rights and fundamental freedoms’. The growing body of literature on supported decision-making points to different ways of formulating this approach – from informal support to a legal framework that recognizes supports in decision-making as a legally valid way to exercise legal capacity (Browning *et al*. 2015). Along with Davidson *et al*. (2015) we emphasize the importance of legal recognition of supports for decision-making in the mental health context; especially if the generic law approach is to be adopted.

This formulation of capacity, referred to as ‘decision-making capability’ (Bach & Kerzner, 2010; Bach, 2011) is informed by Sen's (2004) capabilities approach, recognizing that an effective capability is a combination of unique abilities complemented by goods, services and environments needed to make that ability effective in any particular context.

Bach & Kerzner (2010) propose that the new formula for legal capacity instituted by the CRPD would not do away with legal capacity exercised independently, as measured by the conventional standards for decision-making – which, depending on the jurisdiction, are some version of ability to understand, retain and apply information to the decision at hand, appreciate consequences of one choice over another, and communicate the decision in a manner others can understand. Rather, this should be the presumption in all cases, but that other ways of exercising legal capacity, consistent with the CRPD's ‘support imperative’, should also be recognized. Advance statements could play a significant role in extending a person's legal independence by anticipating a time in the future when the person is unwell and unable to express wishes and have them applied.

Others may not be able to act legally independently at some time because they lack the necessary abilities, and will require supported decision making. Under this approach, people would access the assistance of support persons to turn their expressed desires and intentions into legal acts that respect the person's will and preference, as required under the CRPD.

‘Facilitated decision making’, a third way to exercise legal capacity in this scheme, would be activated when a person is unable to act legally independently; is without support persons able to translate the person's will and preference into decisions – because they are unavailable or because interpretations of the person's will and preferences radically conflict; or the person is expressing wishes and taking actions contemporaneously that are known by others to be in direct conflict with what is known about that person's stable, long-standing and expressed values and wishes. ‘Facilitation’ would entail a person being appointed to mediate a decision-making dialogue with the person, his or her intimate support persons if available, and the professionals. The aim would be to arrive at the ‘best interpretation of a person's will and preferences’ as it would apply in the circumstances, a new standard the UN Committee laid out to address precisely these kinds of situations.

The boundary between these three ways of exercising legal capacity is essential in a liberal-democratic society that respects personal autonomy – and it is precisely this boundary that mental health law struggles with. How do we distinguish between acts that should command the respect of others, and acts violating a person's dignity and causing substantial harm, but avoidable through medical and other supports?

A CRPD-compliant standard for decision-making support and respect for will and preferences would require that a much more nuanced decision-making process than mental health regimes at ‘Level 2’ provide. For example, a simple binary distinction between voluntary–involuntary is superseded. In a mental health system that meets both the ‘generic law’ and the ‘non-discriminatory legal capacity’ standard, a different range of options would need to be available.

This does not mean that non-consensual treatment would no longer have a place. It would, but it would not be triggered by a diagnostic criterion. However, where a person appears to be sustaining significant harms, a different protocol would be activated – outside a medical emergency. The first question is whether a person is acting legally independently? If the person is unable to act legally independently, does a supported decision-making arrangement need to be established, and steps taken for that purpose? If a person is not able to act independently, and supported decision-making is not in place, or has broken down, a ‘facilitated’ process might be required. Non-consensual treatment would only be justified after it is clear that a person is not acting independently, is suffering significant harms, and all efforts to provide support have failed. Such interventions would require additional steps being taken to provide assistance so the person can re-establish their decision-making capability with the expression of their will and preferences.

How could we apply the construct of ‘will and preferences’ in practice? Where a person appears to be having a difficulty with a decision that might carry serious consequences, one would ask: first, ‘what are the person's will and preferences?’ and, second, ‘is the decision consistent with these?’ It would follow that if for any reason, it is not possible to ascertain what the person's will and preferences are, or if there is good reason to believe that the currently expressed will and preferences are inconsistent with the person's ‘authentic’ will and preferences – to which the person has until now shown a deep commitment – then an intervention might be justified, precisely to protect that person's commitments as he or she has expressed them in the past. The aim of the intervention is to give effect as much as is possible to the ‘authentic’ will and preferences. Such a formulation for the justification of non-consensual interventions might indeed prove more satisfactory than one based on our current ideas of ‘decision-making capacity’ and ‘best interests’. It could be argued that facilitating a person's will and preferences at a time when they are unable to do so unassisted and when all attempts at support have failed, is not, in effect, ‘substituted’ decision-making at all [as Bach & Kerzner (2010) have proposed in ‘facilitated’ decision-making, and Szmukler *et al*. (2014) in their development of a generic law].

Of course, ascertaining a person's ‘authentic’ will and preferences may sometimes offer major challenges. The method we advocate is termed ‘interpretation’. We cannot discuss the details here. Essentially it aims at determining a person's deepest commitments, those that express who the person is. Thus unlike a purely ‘value-neutral’ ‘procedural or ‘cognitive’ approach, it considers a person's decision-influencing beliefs and values and whether they ‘cohere’ with those deep commitments. The support of others, beyond a clinical team, especially those who know the person well can be crucial in reaching a ‘best interpretation’ [for a discussion of ‘interpretation’ see Banner & Szmukler (2014); Glover (2014)].

The extent to which autonomy is given pre-eminence of course varies across countries. How would respect for a person's will and preferences fit with such variation? What is being respected are the person's values, beliefs, deep commitments and so on. These will significantly reflect the social and cultural values of the person's world; it would seem consistent with such a values-centred approach that decisions while ultimately ‘owned’ by the person could be made within different relational contexts with different degrees of culturally conditioned sharing in the decision-making process. In all cultures, decision-making often, even usually, takes place within a ‘decision-making community’. Ultimately, though, the vast majority of states have signed up to the CRPD framework and, within interpretative bounds, its version of autonomy.

Sometimes it may not be possible to determine a person's will and preferences in much depth, or with much confidence, for example, where someone has a severe, life-long intellectual disability, who has been institutionalized for many years, and is without those who can play an interpretive role. Under these circumstances a decision might need to be made on the basis of a facilitated approach drawing on whatever threads of understanding about the person's will and preference, and consistent with some notion of ‘human flourishing’; at least until the person has a built a set of personal relationships, in which the person's will and preferences can be more clearly discerned. For the person with a chronic, unremitting, severe psychosis, the question may arise as to whether the person might now have a new ‘authentic’ identity whose values merit respect (Glover, 2014). These are questions that the new legal capacity standard begins to raise, and for which further research and dialogue are required.

## Promising approaches to law reform

What are the prospects for moving towards CRPD-compliant law to achieve the standards we have outlined above? While no regime in the world currently meets the two standards, there is some promise.

We have mentioned the Northern Ireland Mental Capacity Bill in the discussion of the generic ‘fusion’ law standard. Government and civil society organizations have been involved in a series of consultations and reports from mental health advocacy groups indicate strong support for the Bill.

A significant advance on a legal capacity standard is being made in Bulgaria where the national Department of Justice has recently published for public consultation a draft Bill, ‘Natural Persons and Support Measures Act’. This is the result of a government–civil society working group mandated to propose a legal capacity framework following the 2012 judgment of the European Court of Human Rights.^1^ The subject had been deprived of his liberty when placed under guardianship without any right of appeal or review and then admitted involuntarily in a social care home by his guardian on the basis of a mental disorder. The Bill recognizes supports and accommodations for persons to exercise legal capacity in all three ways outlined above – legally independent, statutory supported decision-making status, and ‘facilitated decision making’. This bill does not explicitly address mental health law provisions, but once adopted it would motivate movement towards a fusion approach.

A third example is a draft Mental Health Bill issued by the Zambian Ministry of Justice, prepared following an extensive government–civil society working group process. The objective was to develop a new legislative framework, the first since 1951 that would be CRPD-compliant. The draft bill makes significant progress on the second standard by recognizing the right to legal capacity and the right to access supports for its exercise, and through provision for advance directives, right of review and rights advice. However, it largely retains conventional mental disorder provisions with respect to compulsory treatment.

In conclusion, the CRPD provides a valuable framework and imperative for law reform. Translating the rights recognized in the Convention into the two standards we have proposed gives both civil society groups and governments a common point of reference to guide reform. The examples above show progress is being made on one or the other of the two standards – a generic capacity law, and a regime recognising a range of ways to exercise legal capacity. We recognize that progress depends on many practical issues, the availability of resources being an important one. Especially challenging for many low-income countries is the CRPD Committee's demand that Article 12 – legal capacity – should be realized immediately, not progressively.

We have focused in this paper on legal frameworks for coercive interventions and restrictions of liberty. There has not been the space to consider the equally important requirements under the CRPD for promoting equality of treatment across the whole range of health (including a reduction in the ‘treatment gap’), social, cultural and economic spheres. Nor do we have the space to discuss the changes in power relationships – especially between mental health professionals, families and persons with mental disabilities – that the proposed changes in legal framework would augur.

The law reform efforts outlined here will not, on their own, bring an end to the extensive human rights violations in mental health systems. However, the CRPD-inspired platform and process for reform is giving credibility to the voices and expert knowledge of people with mental health disabilities, and will undoubtedly enhance their standing going forward. This is an essential step in undoing the pervasive stigma and stereotyping that is at the root of the systemic discrimination and violation they face in mental health systems and other sectors.

## Footnotes

† The notes appear after the main text.

1 Stanev v. Bulgaria, App. No. 36760/06, Eur. Ct. H.R. (2012).
